# Possible potential spread of *Anopheles stephensi*, the Asian malaria vector

**DOI:** 10.1186/s12879-024-09213-3

**Published:** 2024-03-20

**Authors:** Qing Liu, Ming Wang, Yu-Tong Du, Jing-Wen Xie, Zi-Ge Yin, Jing-Hong Cai, Tong-Yan Zhao, Heng-Duan Zhang

**Affiliations:** State Key Laboratory of Pathogen and Biosecurity, Beijing Institute of Microbiology and Epidemiology, Beijing, 100071 China

**Keywords:** *Anopheles stephensi*, Malaria, Potential distribution, Biomod2

## Abstract

**Background:**

*Anopheles stephensi* is native to Southeast Asia and the Arabian Peninsula and has emerged as an effective and invasive malaria vector. Since invasion was reported in Djibouti in 2012, the global invasion range of *An. stephensi* has been expanding, and its high adaptability to the environment and the ongoing development of drug resistance have created new challenges for malaria control. Climate change is an important factor affecting the distribution and transfer of species, and understanding the distribution of *An. stephensi* is an important part of malaria control measures, including vector control.

**Methods:**

In this study, we collected existing distribution data for *An. stephensi*, and based on the SSP1-2.6 future climate data, we used the Biomod2 package in R Studio through the use of multiple different model methods such as maximum entropy models (MAXENT) and random forest (RF) in this study to map the predicted global *An. stephensi* climatically suitable areas.

**Results:**

According to the predictions of this study, some areas where there are no current records of *An. stephensi*, showed significant areas of climatically suitable for *An. stephensi*. In addition, the global climatically suitability areas for *An. stephensi* are expanding with global climate change, with some areas changing from unsuitable to suitable, suggesting a greater risk of invasion of *An. stephensi* in these areas, with the attendant possibility of a resurgence of malaria, as has been the case in Djibouti.

**Conclusions:**

This study provides evidence for the possible invasion and expansion of *An. stephensi* and serves as a reference for the optimization of targeted monitoring and control strategies for this malaria vector in potential invasion risk areas.

**Supplementary Information:**

The online version contains supplementary material available at 10.1186/s12879-024-09213-3.

## Introduction

*Anopheles stephensi* is a vector of *Plasmodium vivax* and *Plasmodium falciparum* parasites originating in Southeast Asia and the Arabian Peninsula [1–3]*.* Countries such as India, Afghanistan, Iran, Pakistan, Egypt, Myanmar, Thailand and China are the distribution areas of *An. stephensi* [4]. *An. stephensi* has been implicated in malaria transmission throughout much of its native range in Asia and the Middle East, including India, Iran, and Pakistan [5]. In India, *An. stephensi* is considered an efficient vector of urban malaria [3]. In China, another country with known distribution of *An. stephensi*, the mosquito is not a vector of malaria and has been reported to be distributed mainly in provinces of the oriental realm, such as Guangxi, Hainan, and Sichuan Provinces [6]. However, in recent years, there have been few reports of *An. stephensi* being collected in China [7]. Malaria is a life-threatening disease caused by parasites that are transmitted to humans through the bite of infected female *Anopheles* mosquitoes [8]. There are five parasite species that cause malaria in humans, and two of these species –*P. vivax* and *P. falciparum*– pose the greatest threat [8, 9]. According to the latest World Malaria Report, there were approximately 247 million malaria cases globally in 2021 (245 million in 2020), with an estimated 619,000 malaria deaths, and nearly half of the world's population is at risk of malaria [9]. Moreover, the number of malaria cases reported in Comoros, Costa Rica, Ecuador, Guatemala and some other countries on the mainland shows an increase compared to 2020 [9]. Malaria is considered one of the major vector-borne diseases most sensitive to changes in environmental conditions, similar to other vector-borne diseases such as dengue fever [10, 11]. This is because malaria incidence in endemic areas is largely determined by seasonal variations in mosquito populations and densities [12]. Additionally, environmental factors such as temperature [13] and precipitation [14, 15] can influence malaria incidence by altering the duration of the mosquito and parasite life cycles or by influencing human, vector or parasite behavior [16].

*An. stephensi* is a day-biting, anthropophilic mosquito found mainly in cattle sheds near human dwellings (endophilic) and which feeds indoors (endophagic) [3]. Research on *An. stephensi* in China [6] suggests that it breeds mainly in stagnant water in containers and can also breed in puddles, wells, and pools. Similar to *Aedes* mosquitoes, *An. stephensi* is able to breed in small, artificial containers in urban areas such as domestic water storage containers and garden reservoirs, and it can also breed and develop in larger water-containing structures [17–19] and seems to adapt quickly to local environments (including secluded habitats such as deep wells) [9]. During the dry season, when malaria transmission rates usually reach seasonal lows, *An. stephensi* can withstand extremely high temperatures [9], and this ability of the mosquito to adapt to different environments provides more possibilities for invasion and makes it more challenging to control.

*An. stephensi* is an invasive disease vector, and the evidence is increasing that the geographic range of *An. stephensi* has expanded over the last decade. Invasion by *An. stephensi* was first reported in Djibouti in 2012 [20], Notably, the invasion of *An. stephensi* in Djibouti in 2012 led to a 30-fold increase in malaria cases in Djibouti, from 1,684 cases in 2012 to 49,402 cases in 2019 [9, 19], and a 39-fold increase in malaria cases overall by 2020 [21]. A geostatistical model predicted that the species could spread to many other African cities, which would eventually expose at least 126 million people to risk [22]. Malaria is a major public health problem and challenge worldwide, and as an important vector of malaria, the adaptability of *An. stephensi* to urban environments compared to the main malaria vectors in Africa (*An. gambiae* and *An. funestus*) makes malaria control a greater challenge in more areas [23]. The invasion and population establishment of vector *Anopheles* mosquitoes in new areas brings possible opportunities for malaria transmission, so the invasion of these mosquitoes is also an important threat to global public health.

The shared socioeconomic pathways (SSPs) are new 'pathways' established by a series of international teams of climate scientists, economists and energy system modelers to study possible changes in global society, population and economy over the next century (https://www.carbonbrief.org/explainer-how-shared-socioeconomic-pathways-explore-future-climate-change/. Assessed Sep. 30, 2023). The SSP model is included in the Sixth Assessment Report of the Intergovernmental Panel on Climate Change (IPCC), published in 2021, and has also been used to explore how societal choices will affect greenhouse gas emissions. The SSPs encompass five narratives of the future (SSP1-SSP5), and the SSP1 model describes a world of sustainability-focused growth and equality. By 2100, global carbon dioxide emissions will fall to approximately 22 to 48 gigatons per year (GtCO_2_), and the global surface temperature will rise by 3 to 3.5 °C under the SSP1 climate model (https://www.ipcc.ch/report/ar6/wg1/chapter/chapter-4/. Assessed Sep. 30, 2023). Climate change is an important factor influencing the distribution and transfer of species [24]. Climate change and increased carbon emissions may lead to rapid changes in the global distribution of vector mosquitoes [25], and these changes may even trigger the rapid spread of malaria into more regions.

Vector control is a very effective way to reduce malaria transmission and is an important component of malaria control and elimination strategies. The current research on the global distribution of *An. stephensi* is lacking, and systematic and large-scale surveillance of this vector is still in its infancy [9]. As invasion by *An. stephensi* is intensifying, understanding the current and future global potential areas suitable for the transmission of invasive mosquitoes is important for malaria control and prevention. Species distribution models (SDMs) are valuable and powerful tools for studying the effects of climate change on the potential distribution of species [26–28]. However, uncertainty of SDMs is prevalent due to differences in the use of ecological theories, as well as the assumptions of different SDMs and the different statistical methods used. An individual model may result in differences in suitable habitat for the same species due to multiple factors, leading to uncertainty in predictions [27, 28]. Biomod2 is a new computing framework developed in R for building SDMs [29], exploring the relationship between the spatial distribution of species and environmental variables through the use of multiple different model methods such as maximum entropy models (MAXENT) and random forest (RF), as well as calibrating and evaluating the models, thus improving the accuracy of predicting the potential distribution of species. Therefore, we used the Biomod2 package in R Studio in this study to map the predicted global *An. stephensi* climatically suitable areas, which can provide a necessary tool for assessing the current and future risk of *An. stephensi*.

## Materials and Methods

### Species occurrence data

**Fig. 1:**
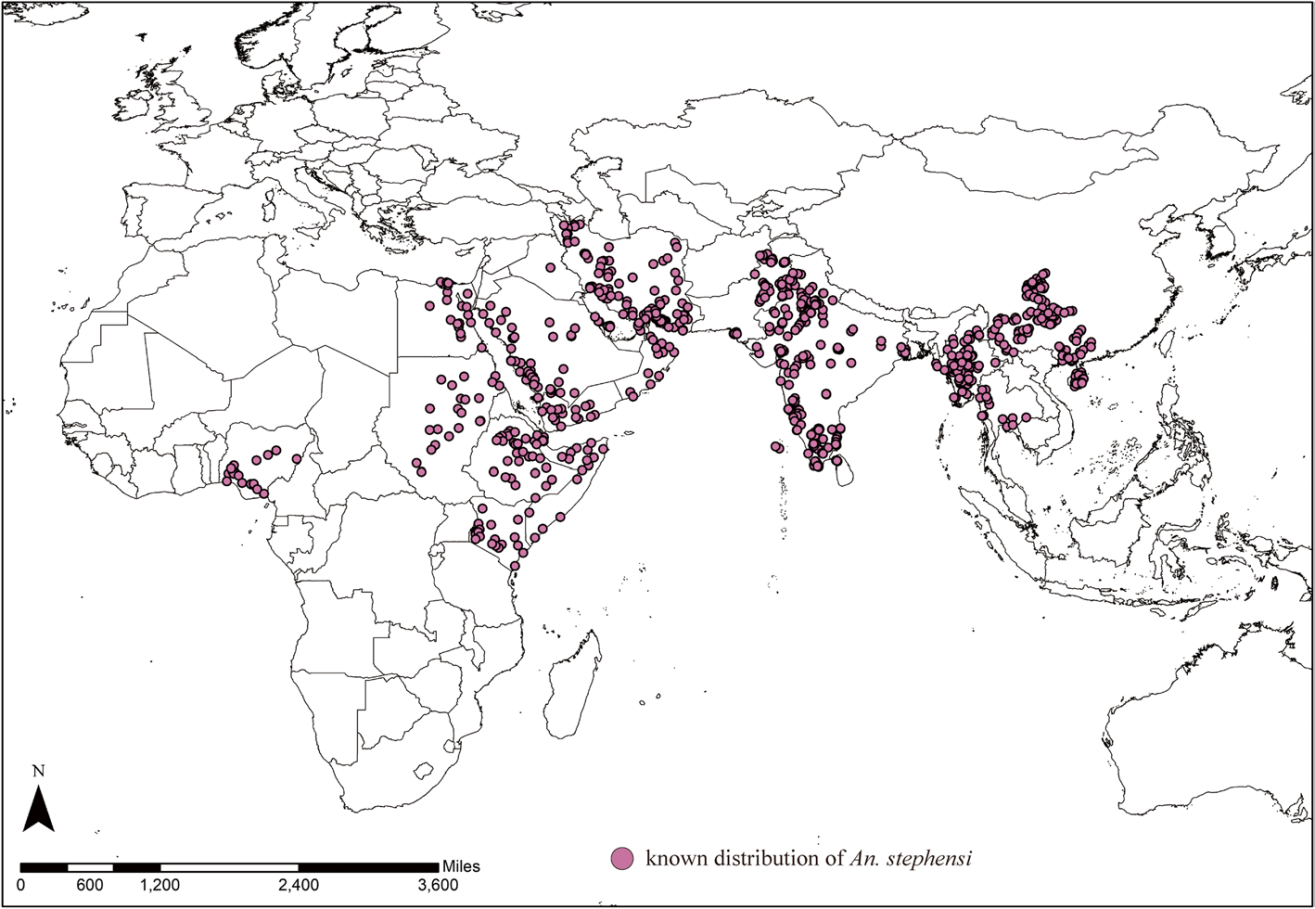
Known global distribution of *An. stephensi*

According to the species occurrence records from the literature (Table S1) and the Global Biodiversity Information Network website (https://www.gbif.org/, GBIF.org, 2019), we selected only those records that clearly originated from human observations, and from these, we screened records with precise latitude and longitude information to ensure geographic accuracy. Ultimately, a total of 964 global occurrence records for *An. stephensi* were obtained for further model construction (Fig. 1, Table S2).

### Current bioclimatic variables

Current bioclimatic variables and elevation data with a spatial resolution of 2.5 m were both downloaded from WorldClim (https://www.Worldclim.org/. Assessed Sep. 16, 2023). The bioclimatic variables contain 19 climate factors (Bio1-Bio19, Table 1), and these variables were divided into 3 categories: (1) 9 temperature-related variables, Bio1, Bio2, Bio3, Bio4, Bio5, Bio6, Bio7, Bioo10 and Bio11. (2) 8 precipitation-related variables, Bio12, Bio13, bio14, Bio15, Bio16, Bio17, Bio18 and Bio19. (3) 2 coupling variables of temperature and precipitation, Bio8 and Bio9. These climate factors are closely related to the distribution of species and are necessary for prediction.

**Table 1:** Environmental variables

Variable	Description	Units
Bio1	Annual Mean Temperature	℃
Bio2	Mean Diurnal Range (Mean of monthly (max temp-min temp))	℃
Bio3	Isothermality (Bio2/Bio7) (× 100)	-
Bio4	Temperature Seasonality (standard deviation × 100)	-
Bio5	Max Temperature of Warmest Month	℃
Bio6	Min Temperature of Coldest Month	℃
Bio7	Temperature Annual Range (Bio5-Bio6)	-
Bio8	Mean Temperature of Wettest Quarter	℃
Bio9	Mean Temperature of Driest Quarter	℃
Bio10	Mean Temperature of Warmest Quarter	℃
Bio11	Mean Temperature of Coldest Quarter	℃
Bio12	Annual Precipitation	mm
Bio13	Precipitation of Wettest Month	mm
Bio14	Precipitation of Driest Month	mm
Bio15	Precipitation Seasonality (Coefficient of Variation)	-
Bio16	Precipitation of Wettest Quarter	mm
Bio17	Precipitation of Driest Quarter	mm
Bio18	Precipitation of Warmest Quarter	mm
Bio19	Precipitation of Coldest Quarter	mm
elev	Elevation	-

To reduce autocorrelation between variable data and avoid overfitting of model predictions, we conducted correlation analyses of environmental factors using maxent.jar software and SPSS 20.0 to calculate Pearson correlation coefficients of the environmental factors before making predictions. Among the two environmental factors with correlation coefficients of | *r* |> 0.9, we chose the one that was more biologically significant. Finally, we selected Bio1, Bio2, Bio3, Bio6, Bio7, Bio8, Bio9, Bio10, Bio12, Bio13, Bio14, Bio15, Bio18, Bio19, and elevation as the environmental factors for modeling

### Future bioclimatic variables

The future bioclimatic variables were also obtained from worldclim (https://www.worldclim.org/). And the bioclimatic variables were under the new set of emissions scenarios Shared Socioeconomic Pathway (SSP) 1–2.6 (https://www.carbonbrief.org/explainer-how-shared-socioeconomic-pathways-explore-future-climate-change/#. Assessed Sep. 16, 2023). A number of these SSP scenarios have been selected to drive climate models for the Coupled Model Intercomparison Project 6 (CMIP6). The data of 2021–2040 and 2041–2060 were selected with the same spatial resolution of 2.5 m in this study.

### Construction and evaluation of model

Ten different model were used in Biomod2, including artificial neural network (ANN) [30], classification and regression tree analysis (CTA) [31], flexible discriminant analysis (FDA) [32], generalized additive model (GAM) [33], generalized boosting model (GBM) [34], generalized linear models (GLM), multiple adaptive regression splines (MARS) [35], maximum entropy models (MAXENT) [36], random forest (RF) [37]and surface range envelope (SRE) [38]. 80% of the distribution records of *An. stephensi* were randomly selected as the training dataset and the remaining 20% as the testing dataset. Kappa coefficients (Kappa) [39], the true skill statistic (TSS) [40] and area under the receiver operating characteristic (ROC) curve (AUC) [41, 42]are used to evaluate the model. Generally, the higher the values of these indicators, the higher the accuracy of the model results [43].

### Construction of ensemble model

The TSS values greater than or equal to 0.8 were selected from all individual models, and two integration methods, committee averaging (CA) and weighted mean of probabilities (WM), were used to integrate these models to produce an ensemble model (EM). Evaluation of the ensemble model produced by the two methods was carried out to select a method that performed better in the model evaluation index. The selected ensemble model was used to predict the potential distribution of *An. stephensi* under future climatic conditions for the years 2021–2040 and 2041–2060, and ultimately to derive a change in the distribution of *An. stephensi* by comparing it with the current climatically suitable areas.

## Results

### Model evaluation

The 10 individual models in Biomod2 were evaluated according to Kappa, TSS and AUC (Table 2). In terms of AUC values, RF has the highest AUC value in all the individual model, followed by GAM and MAXENT. In terms of TSS values, RF, GAM and MAXENT perform better. In terms of Kappa, CTA and SRE results are less than ideal. The best performing models include GAM, MAXENT, and RF models, which have higher values of Kappa, TSS, and AUC when compared to the other models. After selecting models with TSS values higher than 0.8 (GAM, GBM, MAXENT, RF) to construct the ensemble model, the Kappa, TSS and AUC values of the model were improved compared with those of the individual model. In addition, the ensemble model constructed through the WM method had higher Kappa and AUC when the AUC values were almost the same.

**Table 2:** Accuracy evaluation of model

Evaluation index	ANN	CTA	FDA	GAM	GBM	GLM	MARS	MAXENT	RF	SRE	EM(WM)	EM(CA)
Kappa	0.764	0.751	0.793	0.887	0.834	0.788	0.790	0.882	0.962	0.387	0.888	0.829
TSS	0.711	0.700	0.732	0.867	0.837	0.749	0.734	0.907	0.953	0.478	0.924	0.926
AUC	0.901	0.853	0.920	0.986	0.979	0.938	0.917	0.981	0.993	0.739	0.995	0.994

### Current climatically suitable area prediction for *An. stephensi*

**Fig. 2:**
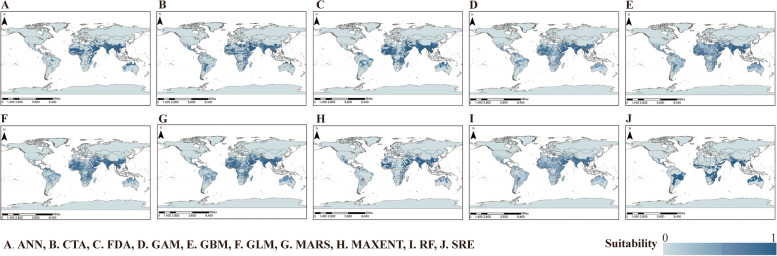
Distribution map of potential climatically suitability areas of *An.stephensi* produced by integrated 10 species spatial distribution model. **A** ANN, **B** CTA, **C** FDA, **D** GAM, **E** GBM, **F** GLM, **G** MARS, **H** MAXENT, **I** RF, **J** SRE

Figure 2 shows the suitability index distribution of climatically suitable areas for *An. stephensi* under the current climatic conditions based on the 10 individual model, and all the data have been normalized. It can be seen from the results that most of the climatically suitable areas of *An. stephensi* obtained from different single models are basically the same, although there are some differences in the distribution areas of *An. stephensi*. Totally, the global distribution of climatically suitable areas for *An. stephensi* is concentrated in parts of Asia and Africa between the Tropic of Cancer (Tropic of Cancer) and the Tropic of Capricorn (Tropic of Cancer), Southern Africa, parts of America and northern Australia.

**Fig. 3:**
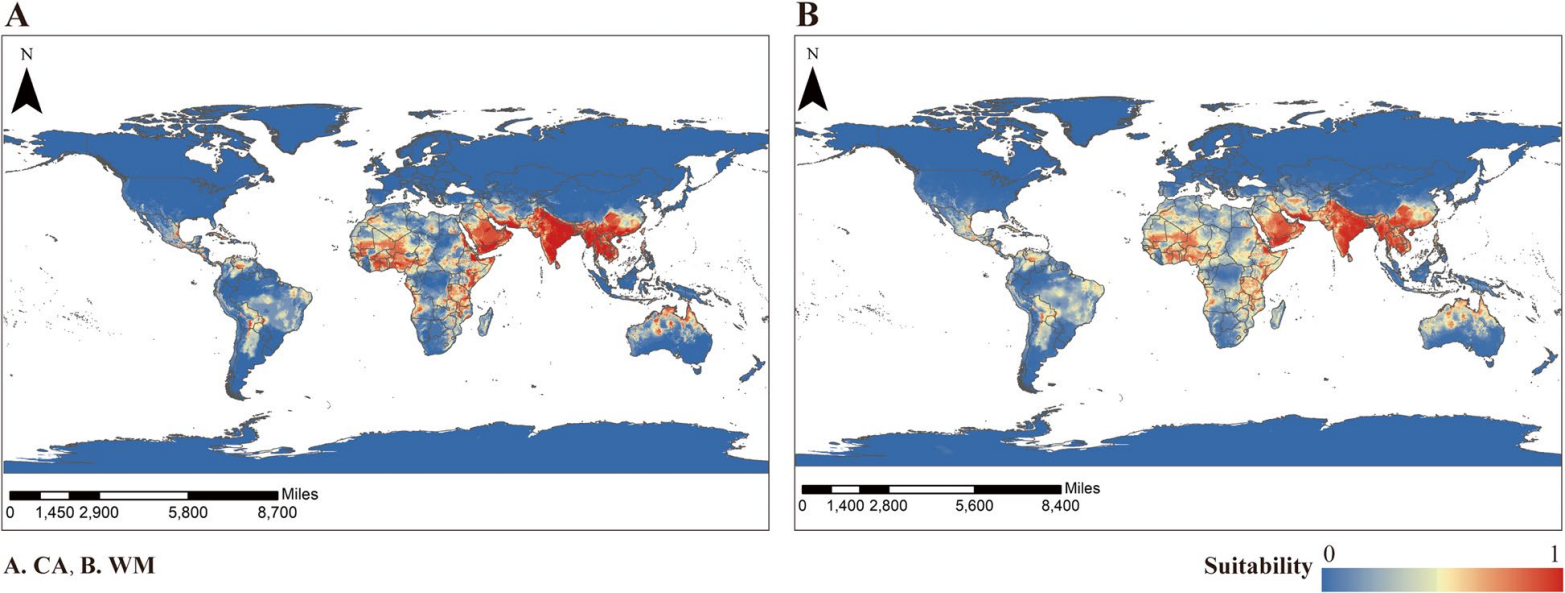
Potential climatically suitability areas distribution of *An.stephensi* predicted by the combined model. **A** CA, **B** WM

The ensemble model (Fig. 3) constructed through the CA and WM methods predicted a similar range of climatically suitable areas for *An. stephensi*. Globally, areas of suitability for *An. stephensi* were concentrated in southern China, India, Pakistan, Afghanistan, Iran and most of Saudi Arabia, Africa, South America and a small portion of the southern United States, and some part of Australia. Overall, areas with a high suitability index for *An. stephensi* were found in most countries in South-East Asia, Saudi Arabia and Africa, particularly in most of the northern part of India bordering Myanmar, Myanmar, Vietnam, the southern coastal areas of China, Saudi Arabia and Algeria.

As can be seen from the model evaluation index (Table 2), the ensemble model constructed through the WM method has a higher Kappa and TSS than the ensemble model constructed through the CA method with the same AUC value, so we chose to use the ensemble model constructed through the WM method when making predictions of the global climatically suitable area of *An. stephensi* in the future climate scenarios.

### Trends of the distribution of climatically suitable areas for *An. stephensi* under SSP1-2.6

**Fig. 4:**
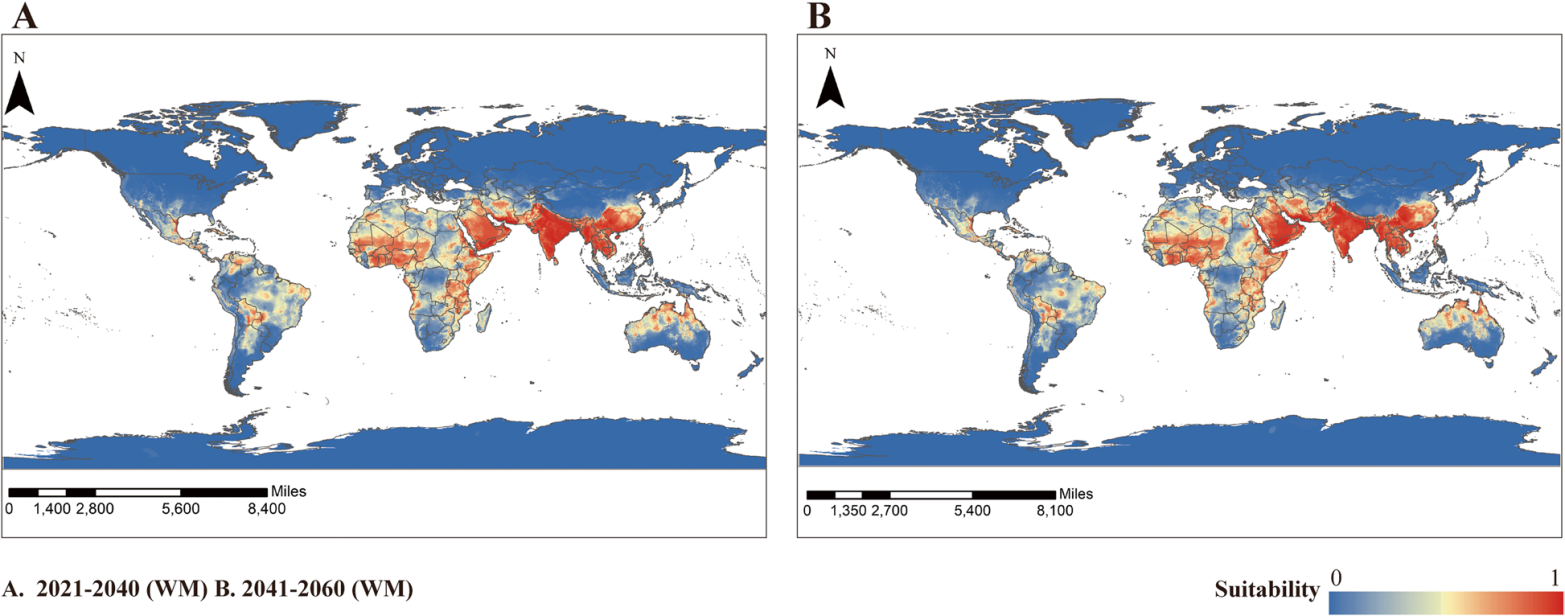
Potential climatically suitability areas distribution of *An.stephensi* in future climate conditions based on ensemble model. **A** 2021–2040, **B** 2041–2060

**Fig. 5:**
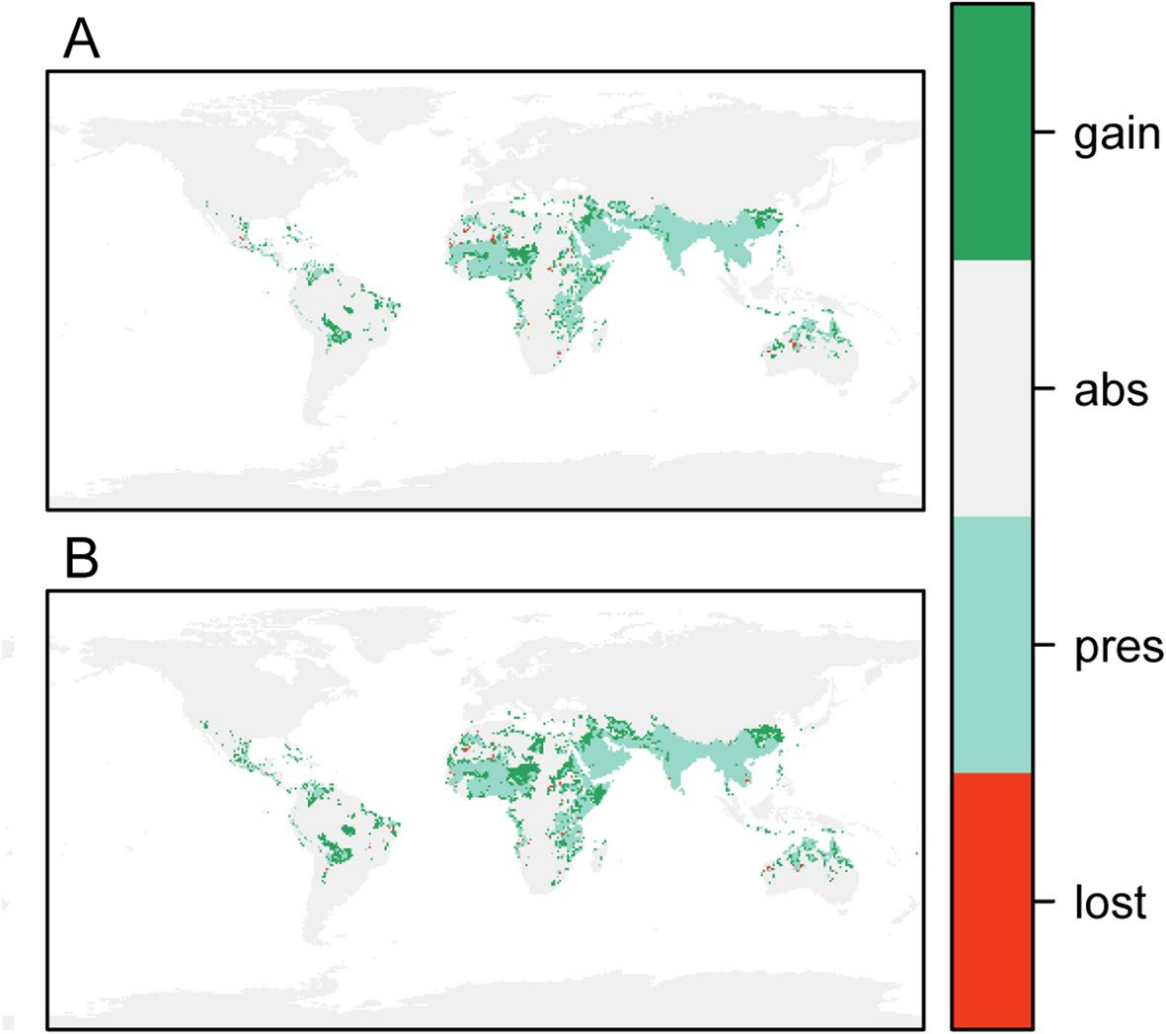
Predicted climatically suitability areas change of *An. stephensi* by ensembled model of WM of probabilities under SSP1-2.6. **A** 2021–2040 **B** 2041–2060

Figure 4-A and B show the global climatically suitable areas for *An. stephensi* in 2021–2040 and 2041–2060, respectively, as predicted by the ensemble model constructed through the WM method based on the SSP1-2.6 climate scenarios. Comparing the results of the ensemble model prediction to the current prediction of climatically suitable areas, the global climatically suitable area for *An. stephensi* will expand by 33.17% from 2021–2040 (Fig. 5-A). From 2041–2060 (Fig. 5-B), the global climatically suitable area for *An. stephensi* will expand by 49.46%. We calculated the percentage of current and future climatically suitable areas for *An. stephensi* predicted by the ensemble model constructed through the WM method, and Table 3 provides a more intuitive view of future trends in the expansion of climatically suitable areas.

**Table 3:** Percentage of climatically suitability areas distribution of *An. stephensi*

Period	Percentage of area (%)	
	**unsuitable**	**suitable**
current	91.59	8.41
2021–2040	88.80	11.20
2041–2061	87.43	12.57

According to our predictions, the global climatically suitable areas of *An. stephensi* is expected to expand and move north-westwards over the next 40 years. The climatically suitable areas of Asia, Africa, America, Australia and other areas for *An. stephensi* are predicted to expand.

## Discussion

The spread of *An. stephensi* is a major potential threat to malaria control and elimination in Africa and southern Asia. The propensity of *An. stephensi* to spread and establish outside the current range is already being observed, drawing the attention of the global health community (https://www.who.int/news/item/29-09-2022-who-launches-new-initiative-to-stop-the-spread-of-invasive-malaria-vector-in-africa. Assessed Oct. 13, 2023). Therefore, it is necessary to better understand the current distribution of *An. stephensi* and the potential future changes in its distribution. Individual SDMs have been used more frequently to predict the potential geographical distributions of invasive mosquitoes [44, 45], while the prediction results using individual SDMs may be under- or over-fitted [43, 46], and ensemble model is well suited to avoid or reduce this uncertainty. Therefore, in this study, we used global distribution data to model the global distribution of *An. stephensi* under current and future climate conditions using Biomod2, provided a map of the potential global distribution of *An. stephensi* under current climate conditions, and predicted changes in the global distribution of *An. stephensi* under future climate change conditions based on the SSP1-2.6 climate scenario. A total of 10 individual models were used in the construction of species distribution models using Biomod2. Among these models, MAXENT is the most commonly used model for species distribution prediction research using individual models [47]. By comparing the prediction results of MAXENT models and ensemble models in this study, we found that in terms of model evaluation indicators, when using the same species distribution data and climatic variables, the ensemble models have higher prediction accuracy. The prediction ranges of MAXENT and ensemble models are essentially the same, though, in terms of predicted climatically suitability areas maps. In addition, according to the prediction results of this study, the best performing models are GAM, MAXENT and RF, which have higher kappa, TSS and AUC values than the other models. These models also have higher predictive accuracy, leading to more accurate simulation effects. The differences in simulation effects between different models may be due to differences in how each model describes the fundamental and realised ecological niches of the species [48]. The ensemble model prediction method constructed in our study is a solution to reduce uncertainty, although it cannot solve the limitations and over-prediction of these individual models themselves, the results of this study show that the ensemble model prediction method is able to improve the model accuracy and reduce the model uncertainty to a certain extent.

Since *An. stephensi* was first reported in Djibouti in 2012, it was collected in Ethiopia and Sudan in 2016 [49, 50], Sri Lanka in 2017 [51], Somalia in 2019 [52], Nigeria in 2020 [53, 54] and Yemen in 2021 [21]. As recently as 2022, *An. stephensi* has also been collected in Kenya [21]. According to the predictions of this study, some areas where there are no current records of *An. stephensi*, especially in North Africa, showed significant areas of suitable habitat for *An. stephensi*. Ahn et al. [54] used bilateral maritime trade data to model and analyze countries with the highest risk of invasion of *An. stephensi* in Africa. The results showed that Djibouti and Sudan are the countries with the highest risk of invasion of *An. stephensi* in Africa, and *An. stephensi* has already invaded and established populations in these two countries. Strong maritime trade links seem to play a positive role in the invasion of *An. stephensi* and help it to invade new areas through human commerce. Countries such as Sudan, Egypt and Nigeria, as the known colonisation location for *An. stephensi*, are the bordering country to Chad, Libya, Niger and Central Africa, which are climatically suitable areas in our predicted results. Trade exchanges between regions are also an important influence on the invasive spread of species. 12 years ago, it might have been unknown to Africa, but 12 years later, *An. stephensi* has nearly invaded and colonised about a third of the African continent. It is justified to assume that these mosquitoes arrived on the African continent as a result of inter-country traffic and trade, accompanied by climate change, which facilitated their settlement. We consider, and our predictions suggest, that *An. stephensi* will spread in the future if no measures are taken.

Many studies have already demonstrated that *An. stephensi* comprises of three ecological variants, namely ‘type’ form, ‘intermediate’ and ‘mysorensis’ which can be characterized by egg morphometry [55–57]. The ‘type’ form is an efficient urban malaria vector in India due to its anthropophilic nature and adaptation to man-made breeding sites, moreover, ‘type’ and ‘intermediate’ forms have also emerged as efficient vectors in rural areas of India as a result of changing agricultural and water storage practices (https://www.who.int/publications/m/item/WHO-CDS-GMP-MPAC-2019.14. Assessed Dec. 13, 2023). But as far as the current research is concerned, we are unable to clearly distinguish these three variants [58]. There is currently no widely accepted molecular identification method for *An. stephensi* [59, 60]. Therefore, there is currently no precise documentation of the occurrence records of *An. stephensi*, as well as its new invasion records in Africa, which can accurately identify the ecological variants of *An. stephensi*. That is, most records of the distribution of *An. stephensi* are now sensu lato, not sensu stricto. We believe that further research is needed to develop reliable and feasible identification methods for these three variants of *An. stephensi*, and to accumulate distribution data for each genotype, in order to provide support for further distribution prediction and vector determination.

*An. stephensi* has been shown to be an effective vector of malaria in both rural and urban areas [61] and has a strong ability to survive and breed in urban areas [2], mainly in water tanks, water storage containers, construction sites, desert coolers, wells, and other artificial habitats [21, 61, 62], potentially placing urban populations at greater risk. In India, *An. stephensi* is the main malaria vector in urban environments and successfully sustains malaria transmission even at low vector densities [63, 64]. Sub-Saharan Africa, the region with the highest malaria burden, with more than 40% of the population living in urban environments [9], has many moderately and lowly suitable areas for *An. stephensi* in our projections, reinforcing the need to strengthen vector surveillance and control in these areas. In addition, the predictions showed that the southern United States, Mexico, and South America have large areas of moderately and lowly suitable habitat for *An. stephensi*. According to the latest WHO Malaria Report [9], Mexico and South America have the highest burden of malaria outside Sub-Saharan Africa, and in the United States, approximately 2,000 malaria cases are diagnosed annually, with the majority of these cases being imported [8]. Thus, once *An. stephensi* has successfully invaded, the suitable environment for its survival will help it to settle and spread in these areas, which will pose a new threat to malaria control. The sensitivity of malaria endemic vectors to insecticides is an important component of developing an effective vector management program [65]. Resistance in *An. stephensi* has been reported in Africa and Asia over the last two years. High resistance to pyrethroids was observed in *An. stephensi* mosquitoes captured in Ethiopia [66], which suggests the limited effectiveness of pyrethroid-only insecticide-treated nets (which have been used throughout Ethiopia) in controlling malaria transmitted by *An. stephensi*. Resistance in *An. stephensi* has also been reported in regions such as Afghanistan [67], Pakistan [68], Dubai [69] and India [70]. The development of resistance in malaria vectors is one of the serious limitations to effective vector control strategies that rely on chemical insecticides [65], and the resistance shown by *An. stephensi* to insecticides in these areas poses an additional challenge to its control. Therefore, to cope with the risk of invasion and malaria transmission associated with the expansion of *An. stephensi* habitats, integrated vector control measures should be actively strengthened in these areas. In terms of protecting populations, attention should be given to improving health education, housing conditions and the use of screens and other barriers to prevent invasive mosquitoes from entering human dwellings, and consideration should be given to adopting or intensifying the use of insecticide-treated mosquito nets or indoor residual spraying. Therefore, in suitable areas where *An. stephensi* has not yet invaded, mosquito species should be routinely monitored, identified and documented to prevent the importation of *An. stephensi*, especially in locations where there is movement of people and trade, such as airports, seaports, land ports and other ports of exit and entry.

The invasive spread of malaria vectors has affected the control programs of many malaria-endemic countries in Africa and Asia [9, 71, 72], posing a great challenge to the control of malaria and its vectors. The predicted results of this study, however, provide evidence for the possible invasion and expansion of *An. stephensi* as well as a reference base for the optimization of targeted surveillance and control strategies for *An. stephensi* in potential invasion risk areas. 

### Supplementary Information

**Supplementary Material 1.**

## Data Availability

The datasets used and/or analyzed during the current study available from the corresponding author on reasonable request.
