# Supplementary material for: Possible potential spread of Anopheles stephensi, the Asian malaria vector
Source: BMC Infect Dis. 2024 Mar 20;24:333. doi: 10.1186/s12879-024-09213-3 (PMC10953274; doi:10.1186/s12879-024-09213-3)
Supplement: Supplementary file 1 — Supplementary Material 1. [file 12879_2024_9213_MOESM1_ESM.zip › supplementary file/Table S3 literature.pdf]

1. Emiru, T., Getachew, D., Murphy, M. et al. Evidence for a role of *Anopheles stephensi* in the spread of drug- and diagnosis-resistant malaria in Africa. Nat Med 29, 3203–3211 (2023). https://doi.org/10.1038/s41591-023-02641-9.

2. Abubakr, M., Sami, H., Mahdi, I., Altahir, O., Abdelbagi, H., Mohamed, N. S., & Ahmed, A. (2022). The Phylodynamic and Spread of the Invasive Asian Malaria Vectors, *Anopheles stephensi*, in Sudan. Biology, 11(3), 409. https://doi.org/10.3390/biology11030409.

3. Hemming-Schroeder, E., & Ahmed, A. (2023). *Anopheles stephensi* in Africa: vector control opportunities for cobreeding *An. stephensi* and *Aedes* arbovirus vectors. Trends in parasitology, 39(2), 86–90. https://doi.org/10.1016/j.pt.2022.11.011.

4. Teka, H., Golassa, L., Medhin, G., Balkew, M., Sisay, C., Gadisa, E., Nekorchuk, D. M., Wimberly, M. C., & Tadesse, F. G. (2023). Trend analysis of malaria in urban settings in Ethiopia from 2014 to 2019. Malaria journal, 22(1), 235. https://doi.org/10.1186/s12936-023-04656-6.

5. Sinka, M. E., Pironon, S., Massey, N. C., Longbottom, J., Hemingway, J., Moyes, C. L., & Willis, K. J. (2020). A new malaria vector in Africa: Predicting the expansion range of *Anopheles stephensi* and identifying the urban populations at risk. Proceedings of the National Academy of Sciences of the United States of America, 117(40), 24900–24908. https://doi.org/10.1073/pnas.2003976117.

6. Balkew, M., Mumba, P., Yohannes, G., Abiy, E., Getachew, D., Yared, S., Worku, A., Gebresilassie, A., Tadesse, F. G., Gadisa, E., Esayas, E., Ashine, T., Ejeta, D., Dugassa, S., Yohannes, M., Lemma, W., Yewhalaw, D., Chibsa, S., Teka, H., Murphy, M., … Irish, S. (2021). An update on the distribution, bionomics, and insecticide susceptibility of *Anopheles stephensi* in Ethiopia, 2018-2020. Malaria journal, 20(1), 263. https://doi.org/10.1186/s12936-021-03801-3.

7. Seyfarth, M., Khaireh, B. A., Abdi, A. A., Bouh, S. M., & Faulde, M. K. (2019). Five years following first detection of *Anopheles stephensi* (Diptera: Culicidae) in Djibouti, Horn of Africa: populations established-malaria emerging. Parasitology research, 118(3), 725–732. https://doi.org/10.1007/s00436-019-06213-0.

8. Al-Eryani, S. M., Irish, S. R., Carter, T. E., Lenhart, A., Aljasari, A., Montoya, L. F., Awash, A. A., Mohammed, E., Ali, S., Esmail, M. A., Hussain, A., Amran, J. G., Kayad, S., Nouredayem, M., Adam, M. A., Azkoul, L., Assada, M., Baheshm, Y. A., Eltahir, W., & Hutin, Y. J. (2023). Public health impact of the spread of *Anopheles stephensi* in the WHO Eastern Mediterranean Region countries in Horn of Africa and Yemen: need for integrated vector surveillance and control. Malaria journal, 22(1), 187. https://doi.org/10.1186/s12936-023-04545-y.

9. World malaria report 2022. Geneva: World Health Organization; 2022. Licence: CC BY-NC-SA 3.0 IGO.

10. Pakdad, K., Hanafi-Bojd, A. A., Vatandoost, H., Sedaghat, M. M., Raeisi, A., Moghaddam, A. S., & Foroushani, A. R. (2017). Predicting the potential distribution of main malaria vectors *Anopheles stephensi*, *An. culicifacies s.l.* and *An. fluviatilis s.l.* in Iran based on maximum entropy model. Acta tropica, 169, 93–99. https://doi.org/10.1016/j.actatropica.2017.02.004.

11. Sinka, M. E., Bangs, M. J., Manguin, S., Chareonviriyaphap, T., Patil, A. P., Temperley, W. H., Gething, P. W., Elyazar, I. R., Kabaria, C. W., Harbach, R. E., & Hay, S. I. (2011). The dominant *Anopheles* vectors of human malaria in the Asia-Pacific region: occurrence data, distribution maps and bionomic précis. Parasites & vectors, 4, 89. https://doi.org/10.1186/1756-3305-4-89.

12. 闫振天,杨飞龙,付文博,李旭东,余果,陈斌.(2013).中国按蚊属昆虫名录修订(双翅目：蚊科).重庆师范大学学报(自然科学版)(06),36-45. Yan, Z.T., Yang, F.L., Fu, W.B., Li, X.D., Yu, G.& Chen, B. (2013). A Revised checklist of Anopheles Species in China (Diptera: Culicidae). Journal of Chongqing Normal University (Natural Science) (06),36-45.

13. 董学书，周红宁. (2019)云南蚊类名录.云南科技出版社, 48-49. Dong, X.S. & Zhou, H.N. (2019) A catalog of the mosquitoes of the Yunnan. Yunnan Science and Technology Press Co., Ltd.
